# Rapid phenotypic stress-based microfluidic antibiotic susceptibility testing of Gram-negative clinical isolates

**DOI:** 10.1038/s41598-017-07584-z

**Published:** 2017-08-14

**Authors:** Maxim Kalashnikov, Marc Mueller, Christine McBeth, Jean C. Lee, Jennifer Campbell, Andre Sharon, Alexis F. Sauer-Budge

**Affiliations:** 1 Center for Manufacturing Innovation, Fraunhofer USA, Brookline, Massachusetts 02446 USA; 2 Division of Infectious Diseases, Department of Medicine, Brigham and Women’s Hospital and Harvard Medical School, Boston, Massachusetts 02115 USA; 3 Department of Mechanical Engineering, Boston University, Boston, Massachusetts 02215 USA; 4 Department of Biomedical Engineering, Boston University, Boston, Massachusetts 02215 USA

## Abstract

Bacteremia is a life-threatening condition for which antibiotics must be prescribed within hours of clinical diagnosis. Since the current gold standard for bacteremia diagnosis is based on conventional methods developed in the mid-1800s—growth on agar or in broth—identification and susceptibility profiling for both Gram-positive and Gram-negative bacterial species requires at least 48–72 h. Recent advancements in accelerated phenotypic antibiotic susceptibility testing have centered on the microscopic growth analysis of small bacterial populations. These approaches are still inherently limited by the bacterial growth rate. Our approach is fundamentally different. By applying environmental stress to bacteria in a microfluidic platform, we can correctly assign antibiotic susceptibility profiles of clinically relevant Gram-negative bacteria within two hours of antibiotic introduction rather than 8–24 h. The substantial expansion to include a number of clinical isolates of important Gram-negative species—*Enterobacter cloacae*, *Escherichia coli*, *Klebsiella pneumoniae*, and *Pseudomonas aeruginosa*—reported here underscores the broad utility of our approach, complementing the method’s proven utility for Gram-positive bacteria. We also demonstrate that the platform is compatible with antibiotics that have varying mechanisms of action—meropenem, gentamicin, and ceftazidime—highlighting the versatility of this platform.

## Introduction

The staggering rise in antibiotic-resistant bacteria combined with the sharp decrease in the development of new antibiotics have combined to create a scenario in which many bacterial infections cannot be treated effectively. Infections resistant to antimicrobials are currently estimated to cause 700,000 deaths each year worldwide^1, 2^; however, a review commissioned to study the growing impact of antimicrobial resistance recently published that this number will likely increase more than 10-fold over the next 35 years “unless action is taken”^3^. Multiple factors are contributing to this rise in antibiotic resistance, including the unregulated use of antibiotics in developing countries and their incorrect use or over-prescription worldwide^4, 5^.

The standard process for determining antibiotic susceptibility for blood-borne pathogens, from patient specimen to final result, takes 48–72 h. This massive bottleneck contributes largely to the widespread prescription of broad-spectrum antibiotics—given the complete dearth of diagnostic information at early time points, attending medical staff have little choice but to prescribe broad-spectrum antibiotics in order to preserve the life of the patient^1, 6, 7^. However, retrospective studies on these empirical prescription practices demonstrate that antibiotics are incorrectly prescribed in as many as 30–50% of cases^8^.

Molecular methods (*e.g*., PCR) for determining antibiotic resistance following positive blood culture exist and can be useful during epidemics. However, these tests are inappropriate for routine diagnosis because 1) susceptibility is not always correlated with genetic markers, 2) genetic markers have not been identified for all antibiotic-resistant bacterial strains^9–11^, and 3) selective pressure leads to a high frequency of genetic mutation^12^. Researchers are working to validate PCR methods on *uncultured* blood. However, limitations of these assays include: the need for ultra-sensitive sample prep, false positives due to circulating DNA or contaminated reagents, false negatives from PCR inhibitors not removed during sample prep, and high detection limits due to the presence of human DNA^13^. Hence, phenotypic assays more accurately determine antibiotic susceptibility.

The main source of the time-lag between collection of the clinical sample and phenotypic susceptibility determination is an issue of scale. When a patient begins to exhibit clinical symptoms of bacteremia, the concentration of bacteria in the blood is on the order of 1–100 colony forming units per milliliter (CFU/mL) in adults^1, 2^ and < 10 CFU/mL in newborns^14^. Therefore, one cannot expect more than 10–1000 bacteria to be present in the standard clinical specimen of 10 mL of whole blood. Because there are also billions of blood cells present in the sample, bacteria must either be purified away from these cells (sample preparation) or multiplied to a much higher concentration (blood culture).

Blood cultures—the current gold standard—take an average of 12–72 h because concentrations of *ca*. 10^8^ CFU/mL (or 20–30 doublings) are required to obtain a positive result^15^. Bacterial species identification following positive blood culture, either through biochemical or molecular assays, guides further antibiotic tailoring and in-depth antibiotic susceptibility testing (AST). According to standard protocols, bacteria are cultured from isolated colonies and propagated to a density of *ca*. 10^8^ CFU/mL prior to AST. Disc diffusion protocols use this high concentration while broth microdilution methods call for 10^5^ CFU/mL^9, 16^. AST results take an average of 24–72 h with manual processing, while semi-automated systems can achieve results within 9–24 h^17–19^.

Microscopy and microfluidics in combination allow for the phenotypic study of individual bacteria or of small bacterial populations in confined volumes^20^. Microfluidic channels handle microliter-sized liquid volumes with microscopic observation routinely possible at the 10-nL scale. At the concentrations routinely used for AST, it would require at most 1000 bacteria, localized in a 10-nL volume, to conduct an experiment equivalent to standard ASTs in a microfluidic setting. Therefore, microfluidic platforms in conjunction with bacterial concentrators^21, 22^ and microscopy can perform the required scaling to close the gap between bacterial levels present in patient samples and those required to perform AST.

Recently, a number of microscopy-based AST platforms have emerged that monitor small bacterial populations^20^. These methods are based on either direct automated counting of bacterial cells^23^, localized density measurement^24^, or observation of metabolite consumption^25^. As such, most of these measurements are a direct function of bacterial growth.

We, and others, are taking a fundamentally different approach by substantially reducing or even circumventing the need for bacterial growth during AST. In these methods, alternative antibiotic susceptibility markers are leveraged to provide much faster results. These alternative markers include fluorescence indicators of bacterial cell death with^26, 27^ or without^28^ stress, monitoring changes in bacterial cell morphology in response to antibiotics^29, 30^, or measuring the change in bacterial vibrations^31^. Our stress-based approach is the only method that pro-actively stimulates bacteria to accelerate their response to antibiotics^20^.

**Figure 1:**
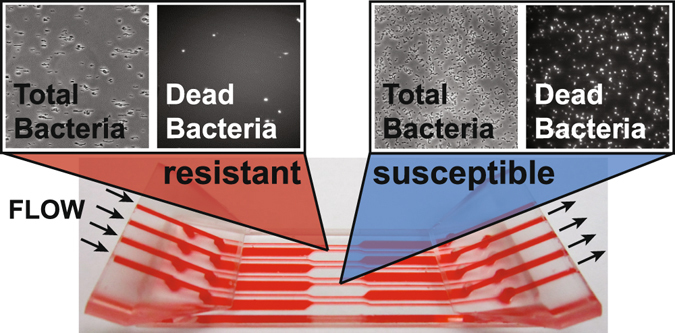
Stress-based antibiotic susceptibility testing method. Shear flow potentiates the action of antibiotics against bacteria covalently-bound to the floor of a microfluidic channel. Bacterial death is monitored over time via automated microscopy. A fluorescent dye gives the number of dead bacteria while phase contrast images show the total bacterial count. If the percent of dead bacteria is >6% at the end of the assay, the strain is susceptible to the antibiotic of interest.

In our rapid AST method, bacteria are covalently bound to the floor of an epoxide-functionalized microfluidic channel, and are subjected to mechanical shear stress created by fluid flowing through the channel (Fig. 1). This mechanical stress potentiates the action of antibiotics, resulting in the rapid cell death of susceptible strains. In contrast, resistant bacteria survive these stressful conditions. Bacterial cell death is monitored via fluorescence using a dead cell stain, and cell death rates are measured for the bacterial samples in the presence and absence of an antibiotic.

Here, we demonstrate the general utility of our platform by testing a number of clinically-relevant antibiotics against Gram-negative bacteria, thereby potently building on prior work with Gram-positive bacterial isolates^26, 27^. Bacterial species were selected to represent four of the most commonly detected Gram-negative species in bacteremic samples: *Enterobacter cloacae*, *Escherichia coli*, *Klebsiella pneumoniae*, and *Pseudomonas aeruginosa*, which collectively are isolated in as many as 40% of positive bacteremia cultures^32^. Selected antibiotics were chosen based on those used as the first line empirical drugs of choice in suspected bacteremia cases at hospitals surveyed in the Boston metro area: meropenem (MEM), gentamicin (GEN), and ceftazidime (CAZ). Inclusion of MEM was particularly important as carbapenem antibiotics are the most frequently administered of all antibiotics for suspected bacteremia cases, but show below 70% efficacy based on follow-up bacterial identification and testing^32^. We note that individual clinics and hospitals administer their own antibiotic stewardship programs based on a diverse array of competing factors and thus the antibiotics selected here may not represent every clinician’s first empirical choice for their patient. However, our selection of antibiotics with different mechanisms of action (beta-lactam and aminoglycoside) highlights the overall utility of our approach. Furthermore, to facilitate future translation to the clinic and to better compare our results with others’, we chose to use clinical breakpoint values for the antibiotic concentrations used in our AST platform^16, 33^.

## Results

### Plasma-assisted assembly of PDMS with pre-coated glass substrate

**Figure 2:**
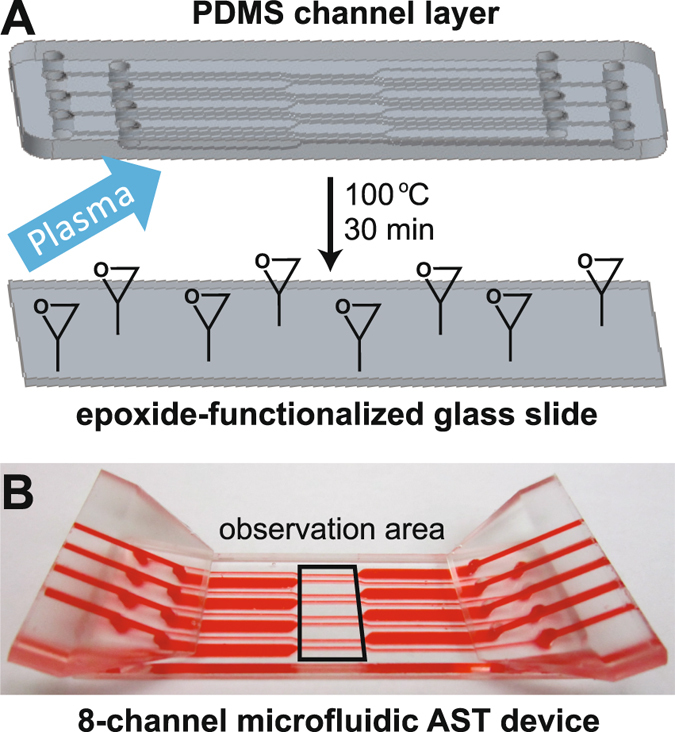
Microfluidic assembly. (**A**) Schematic showing the plasma-assisted thermal bonding of PDMS to an untreated epoxide-coated glass slide. (**B**) Photograph of the assembled microfluidic device. The black rectangle encloses the microscopic observation area.

To interrogate the response of bacteria under stress to antibiotics, it is necessary to immobilize them on the floor of our microfluidic platform. This is achieved by non-specific binding of the bacteria to the epoxide groups on a coated microscope slide. We modified our microfluidic platform^27^ and developed a novel plasma-assisted device assembly protocol (Fig. 2A). New design features included superior device sealing via chemical bonding, a greater number of channels, and improved macro-to-micro fluidic interfacing. Because standard oxygen plasma treatment^34^ would destroy the epoxide coating on the glass surface, we developed a two-step bonding protocol for the device assembly that would be compatible with these reactive groups (see Methods). The bonded device retained the original epoxide coating, was compact, and could reliably sustain leak-free functionality for flow rates greater than 20 mL/min (Fig. 2B).

### Bacterial responses to antibiotics: Changes in fluorescence and morphology

**Figure 3:**
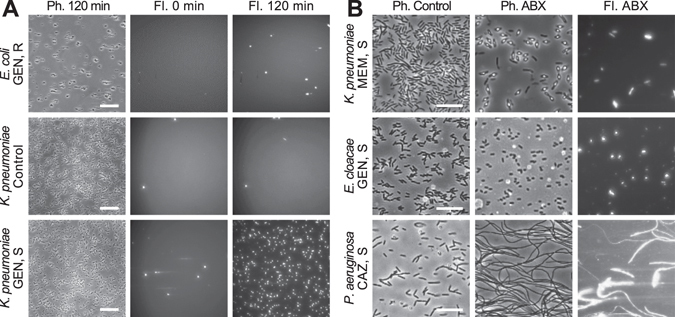
Representative responses to antibiotics of Gram-negative bacteria in our stress-based, microfluidic AST platform. (**A**) Fluorescence responses of GEN-susceptible (*K. pneumoniae* 4453) and GEN-resistant strains (*E. coli* 4456) in the presence (GEN, 2.5 µg/mL) and absence (Control) of GEN. Scale bars are 50 µm. (**B**) Morphological and fluorescence responses of susceptible strains to antibiotics within the flow-cell at t > 30 min. *K. pneumoniae* 426069 forms spheroplasts in the presence of 2 µg/mL MEM, *E. cloacae* 331361 shrinks when exposed to 2.5 µg/mL GEN, and *P. aeruginosa* 4480 fails to divide when breakpoint concentrations of CAZ are present (8 µg/mL). Images are 1/6 of the original field of view. Scale bars are 20 µm.

We collected and processed data from a number of susceptible and resistant strains for each antibiotic: eight susceptible and two resistant to MEM, eight susceptible and four resistant to GEN, and six susceptible and five resistant to CAZ. Qualitatively, we obtained the expected responses in the control vs antibiotic channels of the susceptible and resistant strains. Examples of these responses are shown in Fig. 3A for two Gram-negative bacterial strains against GEN. The cell death of bacteria under shear stress was minimal in the absence of antibiotic after 120 min (Fig. 3A, middle row). However, susceptible strains showed substantially higher levels of death in the presence of fluid shear and antibiotic (Fig. 3A, bottom row). In contrast, resistant strains were able to survive the combination of antibiotic and environmental stresses, and showed minimal increases in fluorescence after 120 min (Fig. 3A, top row).

For different antibiotic/bacteria combinations, we observed significant variations in the cell morphologies of the susceptible strains. This was in accordance with other studies monitoring cell wall changes in Gram-negative strains in response to antibiotics^23^. Representative examples of these changes are shown in Fig. 3B. The observed morphological variations included spheroplast formation for MEM-susceptible *K. pneumoniae* 426069 (Fig. 3B, top row), cell shrinking for GEN-susceptible *E. cloacae* 331361 (Fig. 3B, middle row), and cell elongation for CAZ-susceptible *P. aeruginosa* 4480 (Fig. 3B, bottom row). Only cell elongation as a response to CAZ was a consistent response throughout all susceptible strains, while GEN- and MEM-susceptible strains showed variable morphological responses. Despite the variety of morphological responses, the fluorescence signal consistently increased for all susceptible bacterial strains (Fig. 3B, right column). Therefore, fluorescence was independent of specific changes in cellular morphology as a reaction to an antibiotic, and could be used to quantify cell death effectively and assign susceptibility profiles correctly.

### Time-dependent responses of susceptible and resistant bacterial strains

**Figure 4:**
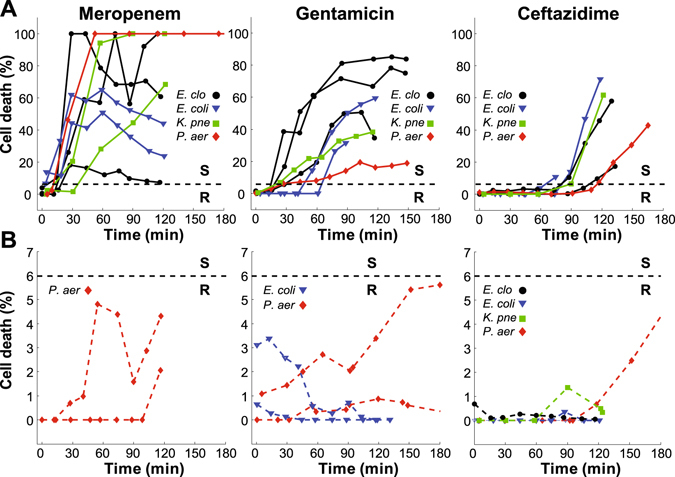
Cell death responses for each Gram-negative strain against different antibiotics. (**A**) Susceptible strains. One experiment per strain shown. The 6% susceptibility threshold is designated with a black line. (**B**) Resistant strains. One representative experiment per strain shown.

Figure 4 shows representative time-dependent data for susceptible and resistant strains to the three antibiotics tested. For clarity, only one experiment per strain was plotted for each antibiotic. In all cases, susceptible strains had sharply rising responses but varied widely in terms of maximum signal (10 to 100%; Fig. 4A). Meanwhile resistant strains exhibited low cell death responses clustering near 1–5% (Fig. 4B). Susceptible strain response times also varied significantly based on the antibiotic. MEM elicited the fastest responses with cell death percentages rising sharply for nearly all strains tested within 10–30 min of initial exposure (Fig. 4, left). Responses to GEN steadily increased from 30–60 min (Fig. 4, middle) whereas bacterial cell death in response to CAZ (at the concentration tested) required between 60–120 min to elicit a response (Fig. 4, right).

From the MEM data, we defined a threshold of cell death percentage that would correctly designate resistant and susceptible strains. The amplitudes of the cell death signal at 60 min of MEM exposure were compared between susceptible and resistant strains. A standard ROC (receiver operating characteristic) curve for threshold steps of 1% yielded two values of 5% and 6% threshold levels as fully separating resistant and susceptible strain results (100% sensitivity and specificity). The larger of the two threshold values was chosen for separating resistant and susceptible strains. Though selection of the 6% threshold was initially based on the MEM data, the same threshold was also appropriate for both the GEN and CAZ data. Experimental results for all of the tested strains are summarized in Table 1. The microfluidic designations (MFD column) were in agreement with minimum inhibitory concentration-based susceptibility assignments obtained using broth microdilution methods (MIC column) in 69 out of 70 experiments. The only disagreement was for *E. coli* 4456, which tested once in our platform as resistant against CAZ. In this case, two additional replicates gave the correct “susceptible” designation.

**Table 1:** Comparison of standard susceptibilities and microfluidic designations obtained using our stress-based AST method. MIC values are given in µg/mL.

	Strain	MEM		GEN		CAZ	
		MIC^a^	MFD^b^	MIC	MFD	MIC	MFD
*E. cloacae*	4431	≤0.3 [S]	S (2/2)^c^	≤1 [S]	S (3/3)	≥64 [R]	R (2/2)
	24214	≤0.3 [S]	S (2/2)	≤1 [S]	S (2/2)	≤1 [S]	S (2/2)
	331361	≤0.3 [S]	S (2/2)	≤1 [S]	S (3/3)	≤1 [S]	S (2/2)
*E. coli*	4149	≤0.3 [S]	ND^d^	≥16 [R]	R (1/1)	16 [R]	R (1/1)
	4456	≤0.3 [S]	S (4/4)	≥16 [R]	R (1/1)	≤1 [S]	S (2/3)
	4674	≤0.3 [S]	ND^d^	≤1 [S]	S (1/1)	≤1 [S]	S (1/1)
	10859	≤0.3 [S]	S (2/2)	≤1 [S]	S (2/2)	16 [R]	R (1/1)
*K. pneumoniae*	4453	≤0.3 [S]	S (2/2)	≤1 [S]	S (2/2)	≤1 [S]	S (3/3)
	426069	≤0.3 [S]	S (3/3)	8 [I]	ND^d^	≥64 [R]	R (1/1)
	434442	–[S]	ND^d^	–[S]	S (1/1)	ND^d^	ND^d^
*P. aeruginosa*	6	8 [R]	R (1/1)	≥64 [R]	R (4/4)	2 [S]	ND^d^
	19	32 [R]	ND^d^	≥64 [R]	R (1/1)	32 [R]	R (1/1)
	4480	≤0.3 [S]	S (3/3)	≤1 [S]	S (3/3)	≤1 [S]	S (3/3)
	361722	–[R]	R (3/3)	–[S]	ND^d^	–[S]	ND^d^

^a^Minimum Inhibitory Concentrations reported in µg/mL.

^b^Microfluidic Designation as determined by cell death; <6% = resistant [R], >6% = susceptible [S].

^c^Number of experiments with stated designation/Total number of experiments.

^d^ND: Not determined.

**Figure 5:**
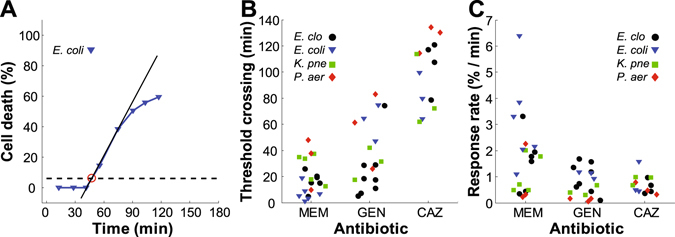
Response dynamics of susceptible strains to the antibiotics tested. (**A)** Cell death percentage of *E. coli* 4674 against GEN. Threshold crossing time (red circle). Linear fit to the rise of the signal (black line). (**B)** Threshold crossing times for each antibiotic. All data are randomly distributed for the same antibiotic. All experimental data are shown. (**C**) Response rates vs antibiotic for all susceptible strain experiments.

The susceptible strains showed quite variable response dynamics against different antibiotics over the observed timescale. Therefore, in addition to setting the binary susceptibility threshold, analysis was conducted to characterize the response dynamics for the susceptible strains. The dynamics were separated into three stages: the rising linear slope of the response, saturation, and a drop in the signal. We analyzed two characteristics common to all three antibiotic data sets: crossing time of the susceptibility threshold (Fig. 5A, red circle) and the rate of response following initiation. The response rate was defined as the slope of a linear fit to the rise of the signal (Fig. 5A, black line). For the example of *E. coli* 4674 with GEN, the threshold crossing time was 47 min and the slope of the linear fit equaled 1.13% cell death per min.

Because each strain was tested at its specific breakpoint concentration, some comparison between the antibiotics is warranted. The summary of the threshold crossing times and response rates are represented in Fig. 5B and C. The figure panels show all of the measured data for each susceptible strain. The number of tested strains of the same species for different antibiotics was insufficient (1 to 3 per antibiotic, see Table 1) to draw statistically significant conclusions about interspecies differences. At the same time, the aggregated data for all species for different antibiotics had clear trends. As expected from qualitative observations, MEM induced the fastest bacterial response (at its breakpoint concentration) with the lowest average threshold crossing time of 19 min. MEM also had the fastest rate of response of 1.8% cell death per min, but the spread in the data was significantly larger than for the other antibiotics. Statistically, 95% of the MEM data crossed the susceptibility threshold within 50 min. Susceptible responses to GEN crossed the threshold within 85 min, while (at the concentration tested) CAZ required 150 min, with the earliest threshold crossing for CAZ occurring at 60 min.

## Discussion

In this work, we have shown that our method cleanly separates and correctly designates susceptible and resistant strains for four species of Gram-negative bacteria against three different antibiotics. The studied antibiotics had two distinct target classes: bacterial cell wall (MEM and CAZ) and protein synthesis (GEN). Our rapid, phenotypic, stress-based method performed equally well in separating resistant and susceptible strains independent of antibiotic class, showing that our method has broad applicability. Our findings also support the view that antibiotics and stress activate common pathways^35, 36^.

AST methods that monitor bacterial growth are hampered by changes in cellular morphology caused by antibiotics. For example, because CAZ causes filament formation of susceptible bacteria (see Fig. 3B bottom row for example), these strains appear to be resistant when based on macroscopic optical density measurements. Even the automated counting of individual bacteria can perform poorly in these circumstances. The importance of including data processing algorithms to correct for these changes, and thereby obtain appropriate designations, was recently reported by Choi *et al*.^23^. In contrast, because we do not measure cell growth but rather cell death by fluorescence, our method is impervious to the diverse morphological changes observed in bacteria that are susceptible to antibiotics. Elongated cells are labeled as efficiently as shrunken cells, eliminating the need for additional processing algorithms, and further demonstrating the merits of our approach.

Here, we selected the antibiotic concentration for testing based on clinical breakpoints to provide yes/no susceptibility assignments. This allowed for the comparison of this novel approach with traditional AST assays used in hospitals and clinics. In traditional assays, clinical breakpoints for specific microbe/antibiotic pairs are compared to the MIC that prevents growth of the bacteria to distinguish resistant from susceptible strains. According to Table 1, the antibiotic concentrations used in our study were 4x–8x the MICs for susceptible strains and ≤0.25x the MIC of resistant strains. Dose response studies will be necessary in the future to establish the connection between the MIC and cell death response, as well as the ability to potentially accelerate separation between susceptible and resistant strain responses using higher antibiotic concentrations. This would be of particular advantage in the case of CAZ where susceptibility assignment at clinical breakpoint concentrations requires approximately 100 min.

We sought to maximize bacterial capture by utilizing a highly reactive generic epoxide chemistry. However, we noted that even with this binding strategy, attachment efficiency varied among clinical isolates. Bacterial strains vary widely in the composition of their outer membrane^36^. Most Gram-negative rods produce a lipopolysaccharide, and many isolates also produce a capsular polysaccharide. The composition of both glycopolymers varies widely among genera and specific strains. Out of the 40 strains that we have tested to date in our platform (including *S. aureus* ^26, 27^, *P*. *aeruginosa*, *E*. *cloacae*, *Klebsiella* spp., and *E. coli*), three did not attach to the surface at the flow rate specified. Whereas all three were clinical isolates of *K*. *pneumoniae*, two other *K*. *pneumoniae* strains readily attached and were given correct susceptibility assignments. Although it was not a parameter in our study, it was later determined that these isolates of *K*. *pneumoniae* were mucoid, reflecting an abundance of capsular polysaccharide. It is likely that the composition or quantity of surface-associated capsule contributed to their poorly adherent phenotype. To date, the bacterial species tested cover approximately 50–60% of the species isolated from bacteremic clinical samples^32^. However, moving forward, expanding the breadth and depth of this study to include additional species and clinical samples will give us a larger view of the scope of attachment variability.

We also note that for AST platforms to reach the clinic, the engineering aspects should have comparable significance to the method development. The assay cartridge must be able to be manufactured on a large scale in order to sufficiently reduce costs for laboratory adoption. Currently, to enable rapid iterative optimization, our device is composed of epoxide-coated glass with PDMS-molded channels. Neither material is compatible with the most common industrial scale processes—injection molding and roll-to-roll manufacturing. To promote ready translation to the clinic, we are developing industrial quality thermoplastic-based devices with epoxide reactivity in-house.

In the future, we see two entry points for our method into the standard clinical workflow. In a conventional clinical workflow, positive blood culture after the initial blood draw is followed by the identification of the pathogen and AST. The method here can be conceptually implemented as is, reducing turn-around times over the semi-automated AST methods currently used. For this, the various aspects of the platform—fluidics, heaters, optics, and microfluidic interface—will need to be incorporated into a single automated instrument. A second, more disruptive clinical workflow would combine our method with rapid bacterial purification directly from blood^21^, circumventing the need for lengthy blood culture. In this case, the bacterial species would not be identified and samples would be tested against a small number of front-line antibiotics used for empirical treatment of bacteremia.

The results reported here were obtained from observing *ca*. 100–4000 bacteria per field of view with most susceptible strain signals reaching 40% cell death. Also, there was no dependence found between the signal amplitude and the initial bacterial surface density of the measured samples. Therefore, conducting AST with bacterial concentrations closer to clinical levels (10–100 CFU/mL or 100–1000 bacteria cells per standard 10 mL clinical blood draw) is theoretically feasible with our method. With the advancement of microfluidic microscopy methods resolving individual bacteria^20^, one can envision a paradigm shift in AST from measuring statistically averaged bacterial populations to directly analyzing clinical samples. From our perspective, methods that require minimal or no growth of the sample and give high bacterial responses would have a substantial advantage in performing AST on minimally propagated samples.

## Methods

### Manufacturing device layers: PDMS channel layer, macrofluidic PDMS adapters

PDMS molds were cut from stainless-steel using an ultra-precision milling machine (UPM-0005, Fraunhofer-IPT, Aachen, Germany). The top surface of the channel mold was finished to below 20-nm roughness using a fly cutting technique to minimize light scattering during microscopic investigation of the samples.

The channel layer was fabricated using standard polydimethylsiloxane (PDMS) (Sylgard® 184, Dow Corning, Auburn, MI) molding processes^26, 27^. Briefly, a 10:1 mixture of curing-agent to PDMS monomer was poured into the mold and thermally cross-linked at 100 °C for 45 min. Each channel layer consisted of eight parallel microfluidic channels. Each channel was 135 µm deep along the whole length, and had variable width from 370 µm in the narrow portion of the channel to 2.5 mm in the wide portion of the channel. The channel layer’s outer dimensions match those of a standard #1 microscope slide.

Macro-to-microfluidic adapters were also cast in PDMS. The adapter ports were designed to overlap the input ports of the channel layer on one end and to accommodate input tubing from the syringe pump on the other. The ports were 1 mm in diameter and, due to PDMS compliance, provided excellent sealing for direct insertion of 1/16” outer diameter FEP tubing (IDEX Health and Science, WA). Also, the slanted adapter design provided stress relief of the tubing and better tube management while placed under the microscope.

The glass substrate (SuperEpoxy2 glass slide, Arrayit Corporation, Sunnyvale, CA) was pre-coated by the manufacturer with epoxide groups for non-specific protein binding of bacterial surface proteins and used as-is.

### Plasma activation of PDMS

To retain the epoxide reactivity on the glass slide, only the PDMS microfluidic layer was plasma-activated (see below). Following surface treatment, the PDMS channel layer was placed on top of an untreated epoxide-coated glass slide, and the two were manually pressed together. This assembly was then placed in an oven at 100 °C for 30 min to bond the materials (Fig. 2A). To complete the device, two PDMS tubing adapters and the PDMS-glass slide assembly were treated with plasma and bonded together at room temperature. In this step, the PDMS channel layer protects the inner-channel epoxide coating from the plasma.

The PDMS channel layer and PDMS microfluidic adapters were treated with oxygen plasma in a plasma surface treatment system prior to bonding (Model PS0500, Plasmatreat USA, Elgin, IL). Parameters were set as follows: base pressure was set to 0.08 Torr, oxygen flow was set to 125 standard cubic centimeters-per-minute (sccm), power was set to 40 W, and the process time was 30 seconds.

### System integration: Phase contrast and fluorescence microscopy

The microfluidic assembly was integrated with an inverted fluorescence microscope (Olympus IX-70, Center Valley, PA) for the acquisition of the fluorescence and phase contrast time-lapse images of bacteria^26, 27^. The XYZ position of the sample was set by an automated XY stage (H117, Prior Scientific Inc., Rockland, MA) and a Z-focus motor (H122R, Prior Scientific Inc., Rockland, MA). The long working distance 60x microscope objective was used for phase contrast and fluorescence imaging (LCPlanFl, Olympus Inc., USA). Both the excitation and emission light passed through a filter cube (U-MWIG, 520–550 nm excitation, > 580 nm emission, Olympus Inc., USA). Each image covered a 250-µm by 250-µm area of the sample. Image data were collected by a monochrome CCD camera (Retiga-4000R, QImaging Surrey, BC, Canada). The data acquisition process was automated using data acquisition controls developed in µManager software^37^.

The media was pumped using a pressure-driven syringe pump (PHD ULTRA pump, Harvard Apparatus, Holliston, MA). A heating module with feedback control was designed and built at Fraunhofer CMI to maintain temperature in the syringes and of the device close to 37 °C. These conditions promoted an active metabolic state of the bacteria for the duration of the AST.

### Bacterial strains and antibiotics

Table 1 lists the 14 bacterial strains comprising four Gram-negative species that were included in the study and showed variable susceptibility profiles to MEM, GEN, and CAZ. *P. aeruginosa* isolates 6 and 19 were isolated in 2011 from patients with catheter and urinary tract infections, respectively, at the University Hospital, Caen, France. MICs for *P. aeruginosa* were determined using standard broth microdilution methods^16^ and cation-adjusted Mueller-Hinton broth. The remaining bacterial strains were isolated between August 2011 and June 2014 from positive blood cultures at Brigham and Women’s Hospital, Boston, MA, and were provided by the Crimson Core facility. MIC values were determined by the hospital clinical microbiology laboratory using the Vitek susceptibility platform and reagents from Biomerieux (Durham, NC). Because the bacterial samples were de-identified before we received them, this study was exempted from human subjects research review.

### Clinical breakpoint selection for antibiotic concentration

Clinical breakpoints for Enterobacteriaceae and *Pseudomonas* species from two of the most commonly used databases were compared: EUCAST^33^ and CLSI^16^. To assign definitive susceptibilities, we selected antibiotic concentrations that matched the clinical breakpoint prescribed for each bacteria/antibiotic combination as shown in Table 2. Where EUCAST and CLSI breakpoints differ, the higher concentration was used (highlighted in bold italics). Due to a four-fold difference in the CAZ susceptibility breakpoints for Enterobacteriaceae species, 2 µg/mL and 4 µg/mL concentrations were used interchangeably. Because o﻿ur gentamicin solid was only 64% active, we ﻿tested this antibiotic at 2.5 μg/mL rather than the intended 4 μg/mL. We note that this difference does not change our findings or their implication﻿s. The same concentration was used to test both susceptible and resistant strains for a specific antibiotic.

**Table 2:** Summary of clinical breakpoint values (given in µg/mL) for antibiotics directed against Gram-negative bacteria. CLSI breakpoints are in parentheses, if different from EUCAST.

EUCAST (CLSI)	MEM		GEN		CAZ	
Sample	S	R	S	R	S	R
Enterobacteriaceae (*Escherichia, Klebsiella, Enterobacter*)	<***2***(1)	>8 (4)	<2 (***4***)	>4 (16)	<1 (***4***)^a^	>4 (16)
P*seudomonas*	<***2***	>8	<***4***	>4 (16)	<***8***	>8 (32)

^a^Both 2 µg/mL and 4 µg/mL concentrations were used.

### Experimental protocol

Bacteria were inoculated from an overnight TSB agar plate into Mueller-Hinton broth (Sigma Aldrich, CA) and cultivated aerobically for 2–4 h at 37 °C to an OD_600_ of 0.2–0.8. This OD corresponded to about 10^8^ to 10^9^ CFU/mL loaded into the device and resulted in a surface density of *ca*. 150 to 4000 bacteria in each 250-μm by 250-µm field of view (12-nL sample volume).

Approximately 70 µL of the bacterial suspension was injected into each channel at the start of the experiment. The microfluidic device was centrifuged for 1 min at 3000 rpm to maximize bacterial attachment. In a single experiment, multiple channels were loaded with the same bacterial suspension and then exposed to the selected antibiotics for various time periods. A single channel with antibiotic-free Mueller-Hinton broth plus 0.5 µM SYTOX Orange (Thermo Fisher, MD) dead cell stain was used as a control for each bacterial strain. Flow rates ranged from 0.1–0.65 mL/min, which corresponded to a shear stress range of 0.8 to 9.3 Pa and shear rates of 1400 s^−1^ to 9600 s^−1^ in the microscopic observation area. These levels of shear stress are comparable to physiologically-achievable levels of shear in blood; with shear rates up to 1500 s^−1^ and as many as 5 Pa of shear stress^38, 39^. Phase contrast and fluorescence images were acquired for each time point at the selected positions along each channel (2–4 positions per channel). Each channel was monitored for 60 to 180 min from the start of the media flow to observe susceptible strain fluorescence for different bacteria-antibiotic combinations. The cycle time in the time-lapse imaging varied due to the variation in the total number of positions and channels used in different experiments, as well as the time necessary for adjustment of small focus misalignments. Precise focus simplified the post-processing image analysis.

### Image analysis

**Figure 6:**
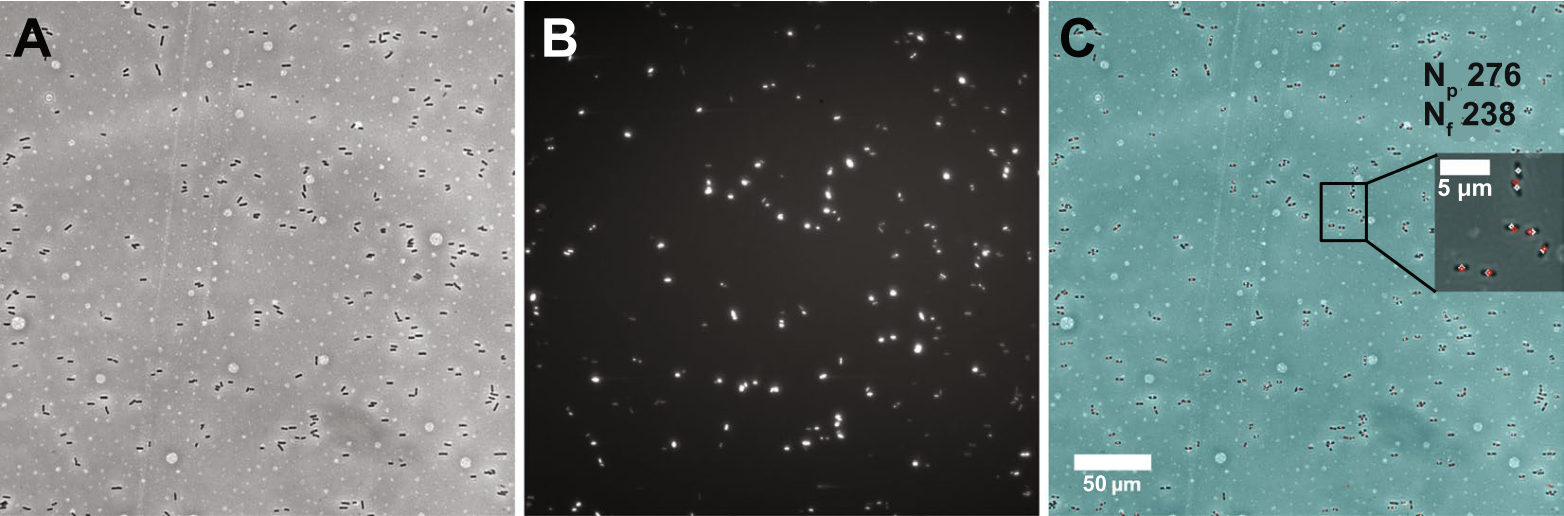
Sample image analysis of GEN-susceptible *E. cloacae*. (**A**) Raw phase contrast image at t = 120 min. (**B**) Corresponding raw fluorescence image at t = 120 min. (**C**) Composite image showing the identified bacteria in the phase contrast (white marks) and fluorescence (red marks) images. N_F_: number of fluorescing cells; N_P_: total number of cells.

The Gram-negative bacteria in the phase contrast images manifested as elongated dark cylindrical particles on a bright background (Fig. 6A). Fluorescence labeling by SYTOX Orange of dead bacteria manifested as bright speckles on a dark background, indicating a high specific affinity of the stain (Fig. 6B). All information obtained from a particular channel position at a specific time point was combined into a single image (Fig. 6C). An original phase contrast image was embedded with markers on each of the counted bacteria (Fig. 6C, insert, white spots) and markers on each of the dead bacteria identified by SYTOX Orange fluorescence (Fig. 6C, insert, red spots). The total counts in the phase contrast and fluorescence images were added to the composite image (N_P_ and N_F_, respectively). Data were normalized to give a measure of bacterial cell death caused by antibiotic alone, and time-dependent traces were then built from these normalized cell death percentage values.

Individual phase contrast and fluorescence images were analyzed using the cell analysis program CellProfiler 2.13 (Broad Institute, Cambridge, MA)^40^. The phase contrast images were inverted to make bacteria bright on a dark background, enhanced for the presence of the tubular structures, thresholded and identified using a maximum correlation thresholding algorithm optimized for neurites^41^. Fluorescence images were analyzed without the inversion, thresholded based on the mode of the image histogram, and individual fluorescing areas were identified^40^. The total counts in the phase contrast and fluorescence images were recorded as N_P_ and N_F_, respectively.

### Time-lapse data analysis

Extracted bacterial count information was further processed using routines developed in Matlab (Mathworks Inc., Natick MA) as previously described^26, 27^. Bacterial counts from an individual fluorescence image (N_F_) were divided by the bacterial counts in the respective phase contrast image (N_P_). The ratio was multiplied by a hundred to evaluate cell death value as a percentage of the total bacteria population. To give a measure of bacterial cell death caused by antibiotic alone, values were normalized by subtracting the percent of dead cells in the control channel from the percent in the antibiotic-containing channel. Time-dependent traces were then built from these normalized cell death percentage values. An empirical threshold was established to separate resistant and susceptible strain data^26, 27^.
